# Dietary replacement of soybean meal with heat-treated fermented soybean meal affects milk production and nitrogen efficiency in lactating dairy cows

**DOI:** 10.5713/ab.250797

**Published:** 2026-03-11

**Authors:** Junsung Kyung, Jaesung Lee, Jinwoo Jeong, Junseok Oh, Kamburawala Kankanamge Tharindu Namal Ranaweera, Sang Yeob Kim, Seyun Im, Hyeonjin Kim, Myunghoo Kim, Myunggi Baik

**Affiliations:** 1 Department of Agricultural Biotechnology and Research Institute of Agriculture and Life Sciences, College of Agriculture and Life Sciences, Seoul National University, Seoul, Korea; 2 AIEcoGenLab Inc., Seoul, Korea

**Keywords:** Crude Protein, Dairy Cows, Milk Production, Nitrogen Utilization, Rumen Undegradable Protein

## Abstract

**Objective:**

The purpose of this study was to see how reducing dietary crude protein (CP) and rumen degradable protein while increasing rumen undegradable protein (RUP) afffected milk yield and composition, nitrogen (N) metabolism, and rumen and blood parameters in Holstein lactating cows.

**Methods:**

Holstein cows (n = 13) were stratified by days in milk (91.75±32.39), parity (2.58±1.44), and milk yield (42.86 kg±6.6), and randomly assigned to one of two dietary groups (soybean meal [SBM] or heat-treated fermented soybean meal [HFSBM] group) in a completely randomized design.

**Results:**

There were no differences in dry matter intake, milk production, or milk composition (fat, protein, lactose, somatic cell count, β-hydroxybutyrate, and milk urea N). Ruminal ammonia concentrations were lower in the HFSBM group than in the SBM group. Ruminal total volatile fatty acid concentrations, acetate and propionate proportions, and blood urea N concentrations did not differ. Calcium levels in the blood were lower both before feeding (0 h) and 3 h post-feeding in the HFSBM group compared to the SBM group. Total protein levels in blood were higher in the HFSBM group. There were no differences in digestibility of dry matter or CP. The neutral detergent fiber and N outputs from feces and urine did not differ. N efficiency tended to be higher in the HFSBM group.

**Conclusion:**

Replacing SBM with HFSBM did not affect milk production but reduced ruminal ammonia concentrations, indicating that reducing dietary CP by increasing RUP levels can be implemented in dairy production without negatively impacting cow performance.

## INTRODUCTION

In modern dairy production, optimizing nitrogen utilization efficiency represents a critical challenge with significant environmental and economic implications. Dairy cows convert only about 25%–27% of ingested nitrogen into milk protein nitrogen, with the remainder excreted through feces and urine [1,2]. This inefficiency can lead to environmental pollution through volatilization, runoff, and leaching of nitrogen [3].

To address these nitrogen losses, determining optimal crude protein (CP) has gained considerable attention. Colmenero and Broderick [4] demonstrated that nitrogen efficiency (NUE) is inversely correlated with the level of dietary CP. Based on a comprehensive meta-analysis of nitrogen utilization data, Huhtanen and Hristov [5] concluded that the most effective nutritional strategies for improving milk NUE in lactating cows were avoiding excessive CP intake and minimizing surplus rumen degradable protein (RDP). In fact, previous research has shown promising results when manipulating dietary protein fractions. In one study, milk yield (MY) was not reduced when CP was reduced from 16.5% to 15.6% [6]. Agle et al [7] reported there was no change in MY when RDP was reduced from 10.3% to 7.1% while rumen undegradable protein (RUP) level was maintained. These findings imply that strategic manipulation of protein fractions rather than total protein content might maintain production while improving NUE.

Soybean meal (SBM) is widely used as a protein source and is known for well-balanced amino acid contents and palatability. Although it contains high levels of protein components, due to its high RDP it is easily degraded in the rumen resulting in inefficient nitrogen utilization. Furthermore, excess ammonia production in the rumen results in higher nitrogen excretion, contributing to environmental pollution. To increase the availability of protein, heat treatment has been introduced [8]. Such treatment modifies protein fractions through induced protein denaturation and structural change, which increases RUP contents. By substituting SBM with heat-treated fermented soybean meal (HFSBM), it may be possible to test the simultaneous reduction of dietary CP and RDP while increasing RUP.

Many studies have investigated reducing dietary CP to mitigate environmental impacts without affecting animal performance. However, few studies have examined the effect of simultaneously reducing CP and RDP while increasing RUP in dairy cow diets. Here, we investigated the changes in performance and nitrogen excretion of lactating Holstein cows due to reduced dietary CP and RDP and increased RUP in feed. We hypothesized that reducing dietary CP and RDP while increasing RUP would not significantly affect MY or milk components with higher NUE.

## MATERIALS AND METHODS

### Animal and experimental design

This research was conducted at a conventional dairy farm located at 37°11′37.4″N, 127°27′45.6″E in Gyeonggi Province, South Korea. The experiment was conducted over 80 days, comprising a 10-day adaptation period followed by a 70-day experimental period. Thirteen Holstein lactating cows were initially used in this study. The cows were in early to mid-lactation (mean days in milk [DIM]±SD: 91.75±32.39 days with an average MY of 42.86±6.6 kg/day and parity of 2.58±1.44). Cows were stratified by DIM, parity, and milk production, and randomly assigned to one of two dietary treatments in a completely randomized design: six cows were assigned to the SBM group, and seven cows were assigned to the HFSBM group. One cow from the SBM group was excluded from the experiment due to health issue, resulting in five cows in the SBM group and seven cows in the SBM group for final data analysis.

Cows were housed in a single pen with a compost-bedded pack. An additional two barns (4 m×5 m) were used to calculate feed intake.

### Experimental diet and feeding trial

Cows were fed the same basal total mixed ration (TMR) ad libitum and had free access to water. The experimental diets differed only in the protein supplement provided. Chemical composition of experimental substrate feed is presented in Table 1, and ingredients and chemical composition of experimental diets are presented in Table 2. Supplements were top-dressed twice daily at 09:00 and 15:00 h. The control group received 1,000 g of SBM per day, while the treatment group received 500 g of HFSBM and 400 g of corn flakes per day. Additional concentrates were provided using an automated feeder system (Delpro, DeLaval) and intake was recorded daily. For feed intake measurements, in each group, cows were housed in individual pens for four consecutive days twice during the experimental period, resulting in a total of eight days of feed intake measurements per cow. Body weights were estimated by using heart girth. Estimated body weights were measured on week 0, 4, and 11. Estimated body weights were used to calculate digestibility, fecal and urine outputs. The equation to calculate estimated body weight is described by Yan et al [9].


(1) Estimated body weight (kg)=6.373 Heart girth (cm)-662

### Fecal and urine sample collection

Fecal and urine samples were collected during weeks 4 and 11 of the study. Feces were collected twice daily via rectal sampling from each animal for three consecutive days, with approximately 300 g per collection. Samples were immediately stored at −20°C for further analysis. After the collection period, fecal samples were composited by equal weight (100 g per cow) and dried in a forced-air oven at 65°C for 72 h. Dried samples were ground through a 1-mm sieve using Wiley Mill (Arthur H. Thomas).

### Fecal and feed analysis

The ground fecal samples, and feed samples were analyzed for dry matter (DM), crude protein, ether extract, ash crude fiber, nitrogen, acid detergent fiber, and neutral detergent fiber (NDF). For feed, additional calcium, magnesium, and phosphorus were analyzed. The DM (method 930.1), CP (Kjeldahl N×6.25, method 981.10), ether extract (method 920.39), and ash (method 942.05) contents of the feed were determined using analytical methods provided by the Association of Official Analytical Chemists (AOAC) [10].

Indigestible NDF (iNDF) was used as an internal marker to calculate estimated digestibility and fecal production. iNDF content of fecal samples, TMR, concentrates, and other feed additives was determined according to Valente et al [11]. Briefly, 1g of dried and ground samples, were weighed in the filter bags (F57; Ankom Technology), and placed into mesh bags, and incubated for 288 h in the rumen from 2 different ruminally cannulated steers. Steers were fed concentrate (ingredients: corn flaked 39%, corn flour 16%, wheat fine 18%, DDGS corn 8%, palm kernel meal 6%, soy hulls 5%, limestone 1%, molasses 2%, lysine 2%, copra meal 0.8%, and vitamins and minerals 2%) and oat hay twice daily. After incubation, filter bags were rinsed until the water was clear. All the samples were dried in a dry-oven 65°C for 48 h before iNDF analysis. The NDF were analyzed using a sequential method in an ANKOM200 fiber analyzer (Ankom Technology). Urine was collected at the same time with fecal sample collection. Urine was collected by gentle stimulation of the perineum each sampling. To prevent the N loss in the urine, 50 mL aliquots of urine was added to 0.3 mL of 4N H_2_SO_4_. Urine samples from each sampling were composited by equal volume to prepare one period per animal. The urine samples were analyzed for N content (AOAC International [12]; method 990.13), and creatinine was measured using the Cobas c311 analyzer (Roche).

### Nitrogen excretion and digestibility calculations

The formulas used to calculate iNDF, digestibility, and total fecal output were described in Adams et al [13]. The iNDF was calculated using the following equations.


(2) iNDF (% DM)=P1 (100-P2[%])

Where P1 is NDF % of the sample after equation, and P2 is loss% of sample after incubation.

The digestibility and fecal excretion of DM were calculated using the following marker equations:


(3) DM digestibility (%)=100-(Feed iNDF [%]/Fecal iNDF [%])×100

(4) Fecal DM (kg)=DM intake (kg)×(1-DM digestibility)

(5) Fecal NDF (kg)=NDF intake (kg)×(1-NDF digestibility)

Urine output was estimated from urine creatinine concentrations obtained from spot samples using following creatinine coefficient [14,15]:


(6) Daily creatinine output (mg)=20 mg/kg of BW per day

(7) $$\begin{array}{l} \text{Urine output }(\text{kg}/\text{day})=(29\;\text{mg}/\text{kg of BW per day})\times \\ \text{BW }(\text{kg})/(\text{Urine creatine concentration }[\text{mg}/\text{dL}]\times 10) \end{array}$$

where, 10′ was used for converting ‘dL’ to ‘Liter’.

Total excretion of urinary N was calculated by multiplying estimated urine outputs by the corresponding N concentrations.

### Milk yield and composition analysis

Cows were milked twice daily, and individual MY was recorded by 2×8 DeLaval herringbone parlor HB50 (DeLaval). Milk samples were collected at weeks 0, 2, 4, 6, and 11. Samples were collected for both morning and evening milkings, pooled and were analyzed for fat, protein, lactose, somatic cells, beta-hydroxybutyrate in milk (BHBm), milk urea nitrogen (MUN), solid non-fat (SNF), and total solid (TS) by Bentley Combi 150 (Bentley Instruments). Additionally, corrected MYs were calculated.


(8) $$\begin{array}{l} \text{Fat corrected milk }(\text{FCM})=\\ 0.4\times \text{Milk yield}+16.23\times \text{Fat yield }(\text{kg}/\text{d}) \end{array}$$

(9) $$\begin{array}{l} \text{Energy-corrected milk }(\text{ECM})\;(\text{kg}/\text{d})=\\ 0.323\times \text{Milk yield }(\text{kg}/\text{d})+12.82\times \text{Milk fat yield }(\text{kg}/\text{d})+\\ 7.13\times \text{Milk protein yield }(\text{kg}/\text{d}) \end{array}$$

(10) $$\begin{array}{l} \text{Fat-protein-corrected milk }(\text{FPCM})=\\ (0.377+0.116\times \text{Milk fat }[\%]+0.06\times \text{Milk protein }[\%])\times \\ \text{Milk yield }[\text{kg}/\text{d}] \end{array}$$

### Blood collection and analysis

Blood samples were collected from cows at three time points: week 0 (adaptation period), week 4, and week 11. Samples were taken before feeding (0 h) and 3 h post-feeding. Blood was drawn from the jugular vein using three 10 mL non-heparinized vacutainers (BD Biosciences) for serum preparation. The blood samples were stored in an icebox during collection. To obtain serum, the samples were centrifuged at 2,500×g for 15 minutes at 4°C. The resulting serum aliquots were stored at −70°C for later analysis. Serum samples were analyzed for the concentrations of albumin, alanine aminotransferase (ALT), aspartate aminotransferase (AST), glucose, blood urea nitrogen (BUN), total protein, calcium, magnesium, and inorganic phosphate. The analysis was commissioned by Global Clinical Central Lab (GCCL) and performed using an automatic analyzer (Cobas 6000; Roche).

### Rumen fluid collection and analysis

Rumen fluid was collected using an oral stomach tube 3 h after feeding during weeks 0, 4, and 11 as described by Shen et al [16]. The samples were strained through cheesecloth for pH, volatile fatty acid (VFA), and ammonia analysis. The pH of the rumen fluid was measured immediately using a pH meter (Ohaus). For VFA analysis, 1 mL of rumen fluid was mixed with 0.2 mL of 25% metaphosphoric acid and stored at −70°C. For ruminal ammonia nitrogen (NH_3_-N) analysis, 1 mL of rumen fluid was dispensed and stored at −70°C. Ammonia concentrations were determined using a modified colorimetric method [17]. VFA concentrations were analyzed by gas chromatography using an Agilent Tech 7809A (Agilent Technologies) with a Supelco fused silica capillary column (SUPELCOWAX 10 Capillary GC Column).

### Temperature-humidity index

Data of average environmental temperature (°C) and relative humidity (%) were obtained from the Korea Meteorological Administration for the experimental period [18]. The study was conducted in Moga-myeon, Icheon-si, Gyeonggi-do, Republic of Korea. Temperature-humidity index (THI) was calculated based on the equation described by Dikmen and Hansen [19]:


(11) THI=(1.8×T+32)-([0.55-0.0055×RH]×[1.8×T-26])

Where T is the environmental temperature (°C) and RH is the relative humidity (%).

### Statistical analysis

The data were analyzed using a mixed model by PROC MIXED of SAS (ver. 9.4; SAS Institute). The statistical power (1−β) of the study was calculated post hoc using G*Power 3.1.9.7, based on the given sample size (n = 12), α=0.05, and effect size (f = 0.24), resulting in an estimated power of 76.6%. The model included dietary treatment, period, and their interaction as the fixed effects and animal as a random effect. Baseline (day 0) values were included as covariates in the mixed model analyses to control for initial differences among animals. Data were checked for normality using the UNIVARIATE procedure before analysis. Five variance-covariance structure (Autoregressive type I, Compound Symmetry, Unstructured, Toeplitz, and Variance Components) were tested, and the covariance structure that minimized the Schwarz’s Bayesian information criterion was chosen. Data were also analyzed using the t-test to examine differences between groups. The least-squares means, and corresponding standard error of the mean values were calculated and presented. All the table values were shown by the least squares mean and standard error of the mean value provided by the procedures of SAS. Statistical significance was regarded at p≤0.05 and tendency at 0.05<p≤0.10.

## RESULTS

### Nutrient intake and milk production

Both the SBM and HFSBM diets were formulated to provide the same net energy for lactation (NE_L_) of 1.69 Mcal/kg of DM (Table 2). Dietary CP content was reduced from 16.9% to 16.2% of DM. RDP decreased from 10.96% to 10.1% of DM, while RUP increased from 5.91% to 6.14% of DM. This reduction in dietary CP showed no effect on feed intake, and CP, RDP, and RUP intakes did not differ (Table 2).

No differences between diets were observed in MY, FCM, ECM, and FPCM, nor in the feed efficiencies of these measures (Table 3). No changes were observed in milk composition parameters including fat, protein, lactose, somatic cell count (SCC), BHBm, SNF, MUN, and TS.

**Figure 1:**
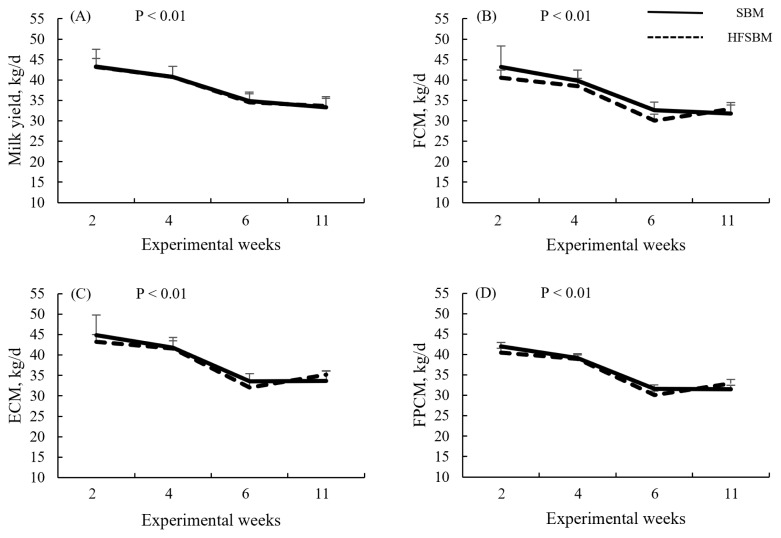
Effects of dietary replacement of soybean meal with heat-treated soybean meal on (A) milk yield, (B) fat-corrected milk (FCM), (C) energy-corrected milk (ECM), and (D) fat-and protein-corrected milk (FPCM) in lactating Holstein cows. Solid lines (—) represent soybean meal (SBM) group, and dashed lines (-----) represent heat-treated soybean meal (HFSBM) group. Error bars represent the standard error of the mean. P values represent period effect, and no treatment effects were found.

**Figure 2:**
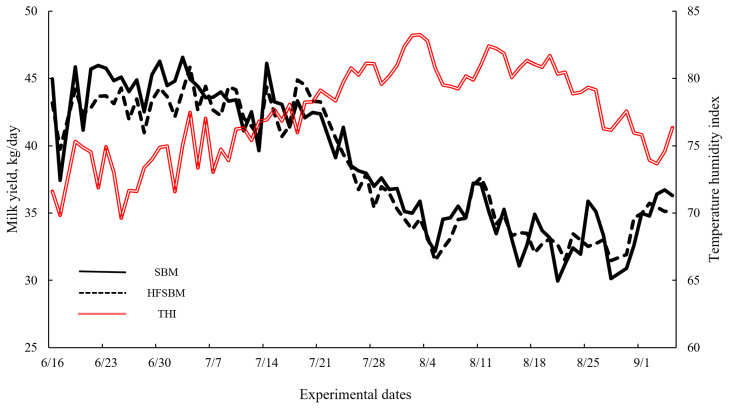
Effects of temperature humidity index (THI) and milk yield over the experimental period. SBM, soybean meal; HFSBM, heat-treated fermented soybean meal.

MY, FCM (p<0.01), ECM (p<0.01), and FPCM (p<0.01) were reduced over the period (Figure 1). Feed efficiencies of MY (p<0.01), FCM (p = 0.05), ECM (p<0.01), and FPCM (p<0.01) were affected by the period. The percentage of milk fat (p = 0.01), and protein (p<0.01) increased. Weights of milk fat (p<0.01), protein (p<0.01), lactose (p<0.01), MUN (p<0.01) were reduced over the period, while SCC increased over the experimental period (p = 0.03). A negative relationship between the THI and lactational responses was observed (Figure 2).

### Rumen fermentation

Ruminal pH, ammonia, and VFA concentrations did not differ between treatments (Table 4). The acetate-to-propionate ratio increased in the HFSBM group, while it decreased in the SBM group over the experimental period (p = 0.02). Treatment × period interactions (p<0.01) were observed. No differences were detected in VFA proportion including acetate, propionate, isobutyrate, butyrate, isovalerate, valerate, and concentration of total VFA production.

### Blood metabolites

**Figure 3:**
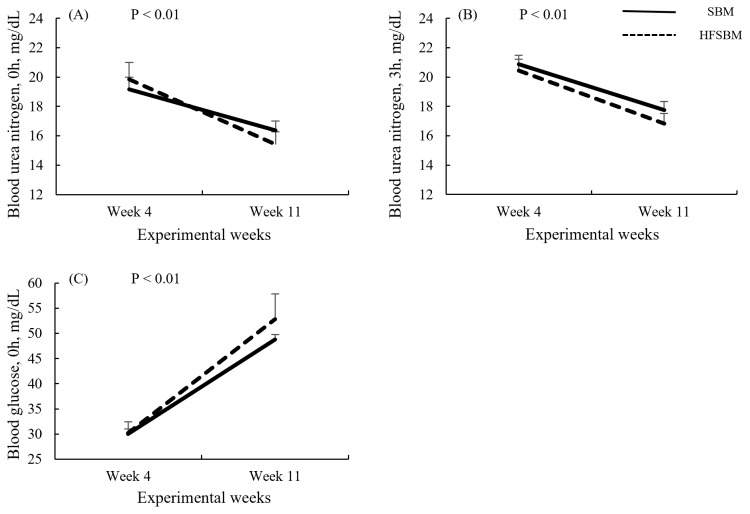
Effects of dietary replacement of soybean meal with heat-treated soybean meal on (A) blood urea nitrogen before feeding (mg/dL) (B) blood urea nitrogen 3 h after feeding (mg/dL), and (C) blood glucose level before feeding (mg/dL) in lactating Holstein cows. Solid lines (—) represent SBM, and dashed lines (-----) represent HFSBM. Error bars represent the standard error of mean. SBM, soybean meal; HFSBM, heat-treated fermented soybean meal. P values represent period effect, and no treatment effects were found.

Blood calcium was higher in the HFSBM group for both before feeding and 3 h after feeding (p = 0.02, p = 0.03, respectively). Total protein concentration 3h after feeding was higher (p = 0.03) in the HFSBM group than in the SBM group. No differences were found in albumin, ALT, AST, glucose, BUN, phosphate, and magnesium concentrations (Table 5). Blood glucose concentration before feeding (0 h) increased (p<0.01), and BUN concentrations decreased both 0 h and 3 h after feeding (p<0.01) over the experimental period (Figure 3).

### Digestibility and nitrogen metabolism

**Figure 4:**
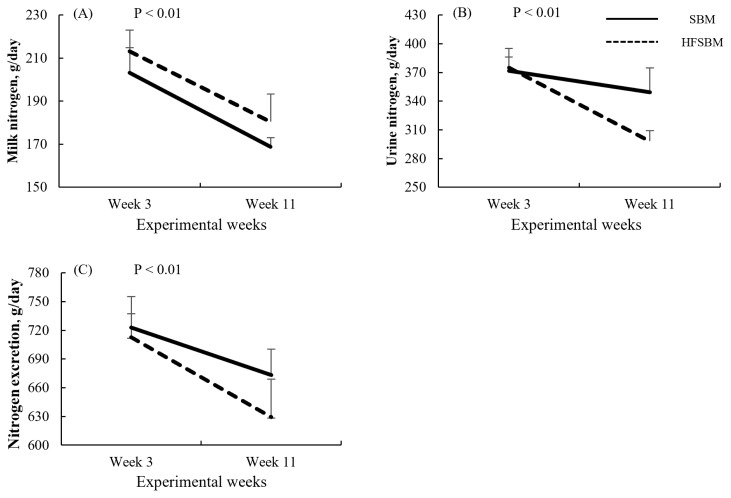
Effects of dietary replacement of soybean meal with heat-treated soybean meal on (A) milk nitrogen (g/d), (B) urine nitrogen (g/d), (C) nitrogen excretion (g/d) in lactating Holstein cows. Solid lines represent SBM, and dashed lines represent HFSBM. Error bars represent the SEM. SBM, soybean meal; HFSBM, heat-treated fermented soybean meal; SEM, standard error of the mean. P values represent period effect, and no treatment effects were found.

No differences were observed between treatments in nutrient digestibility parameters, including DM, CP, and NDF (Table 6). Nitrogen intake, fecal output, and urine volume remained similar between treatments. Nitrogen excretion including urinary, fecal, and milk nitrogen showed no differences. However, NUE tended to be higher (p = 0.08) in cows in the HFSBM group compared to the SBM group. Urinary creatinine concentrations were also similar between treatments. Milk N (p<0.01), urine N (p<0.01), and N excretion (p = 0.01) decreased across periods (Figure 4).

## DISCUSSION

### Nutrient intake and milk production

Reducing dietary CP and RDP levels while increasing RUP through replacement of SBM with HFSBM did not affect dry matter intake (DMI). This is consistent with Bahrami-Yekdangi et al [6], who reported no changes in DMI when dietary CP was reduced from 16.5% to 15.6% with RDP decreasing from 10.9% to 10.1% while maintaining RUP at 5.5% on a DM. Similarly, MY, ECM, FCM, and FPCM were unaffected. These results align with Kalscheur et al [20], who reduced RDP from 9.6% to 8.2% while RUP was fixed at 5.8%. They confirmed that there was no difference despite the RDP being reduced. This implies that milk production can be maintained by reducing the RDP when the RUP is adequately supplied.

In this study, period effects were observed for MY, ECM, FCM, and FPCM (p<0.01) throughout the experimental period. Average MY declined from 43 kg to 33 kg between week 2 and week 11, with corresponding similar decreases observed in ECM, FCM, and FPCM. The experiment was conducted during summer (June to September) and the THI ranged between 72 and 83. An inverse relationship between THI and MY was evident, consistent with West [21], who reported a linear reduction in milk production as THI increased. The THI threshold for lactating cows producing more than 35 kg/d is 68 [22], indicating that the cows in the current study experienced heat stress throughout the experimental period. Furthermore, Kadzere et al [23] demonstrated that high-producing cows experience more heat stress; our results support this, as the initial average milk production was 40.47 kg/d. The heat stress conditions during the experiment may have masked potential diet-induced differences, emphasizing the need for future trials under thermoneutral conditions or in cooler seasons to isolate better dietary protein effects on lactation performance in normal conditions.

Milk composition remained unaffected by the dietary treatments in the current study, consistent with Bahrami-Yekdangi et al [24], who observed similar results when reducing dietary CP from 18.6% to 15.6%. However, they found that MUN responded linearly to CP reductions, although no differences were observed between CP levels of 17.2% and 16.4%. Davidson et al [25] observed no significant changes in milk fat and protein when varying RUP levels (34%, 40%, and 46% of CP) while maintaining consistent CP levels (16.5%–16.8%). However, MUN concentrations were consistent RUP levels. Similarly, Campanile et al [26] found no differences in MUN levels in lactating buffaloes with RDP intake of 1,992 g vs 1,173 g. In contrast, Kalscheur et al [20] reported a linear reduction in MUN as RUP decreased from 11.0% to 6.8%. The lack of MUN response in the current study implies that the difference in CP levels between SBM and HFSBM diets was minimal. Broderick [27] reported a lower milk protein percentage when dietary CP decreased from 16.7% to 15.1%. This contrasts with our findings, where CP decreased from 16.9% to 16.2%, representing a smaller reduction. The smaller decrease in CP in our study may explain the lack of observed differences in milk protein percentage and MUN production.

Throughout the experimental period, milk fat and protein concentrations increased even as their absolute yields declined. Previous research has demonstrated that an increasing THI typically decreases milk fat and protein contents [28,29]. The apparent contradiction observed in our study can be explained by the SCCs, which increased as the THI increased. This aligns with Nasr and El-Tarbany [30], who reported that SCC increased from 216×10^3^ cell/mL to 259×10^3^ cell/mL between THI levels of medium (70–80) and high (>80).

### Rumen fermentation characteristics

Ruminal pH, ammonia, and VFA concentrations were unaffected by dietary treatments in the current study. These findings align with previous research demonstrating similar results under comparable CP reduction. Oh et al [31] reported no changes in ruminal pH and ammonia when CP was reduced from 16.5% to 15.5%, implying that modest CP reductions may not substantially alter rumen fermentation parameters. The reduction in CP from 16.9% to 16.2% in our study falls within this range of minimal change, supporting these observations. Even greater reductions in CP did not affect these ruminal parameters. Bahrami-Yekdangi et al [24] observed no differences in ruminal pH and ammonia concentrations when dietary CP decreased from 18.6% to 15.6%. Regarding VFA concentrations, Agle et al [7] found no differences when dietary CP was reduced from 15.4% to 13.4%; our results are consistent with those findings despite their larger reduction in CP.

### Blood metabolites

Blood calcium concentrations were higher in cows fed HFSBM compared to those fed SBM, both before and at 3 h after feeding. The process of producing HFSBM involves fermentation before it is heat treated. During fermentation, antinutritional factors such as trypsin inhibitors and phytic acid are reduced [32,33]. In an experiment conducted on lactating sows, Zhe et al [34] reported substituting SBM with fermented SBM significantly increased the coefficient of apparent total tract digestibility of ash. This suggests that the fermentation process may enhance mineral bioavailability by neutralizing mineral-binding inhibitors like phytic acid. While direct calcium digestibility was not measured in the current study, the elevated blood calcium levels in the HFSBM group likely reflect this improved nutrient utilization.

BUN concentration remained unaffected by treatments, contrary to our expectations. BUN is directly linked to ruminal protein degradation, in which microbes break down protein into ammonia, which is absorbed through the rumen wall and converted to urea in the liver. This urea then circulates in the blood and is partially transferred to milk as MUN [17]. We assume that the lack of significant differences in BUN concentrations between the treatments may have been due to the small differences in dietary CP levels between the diets. As dietary CP content is a primary driver of ruminal ammonia production and subsequent blood urea formation, the relatively comparable CP levels in our treatments probably resulted in no significant differences in BUN concentrations.

Blood glucose increased from weeks 4 to 11. Glucose is the main precursor of lactose [35], and about 20% of glucose is converted to lactose during lactation [36]. Therefore, high-producing cows require higher glucose levels. However, heat stress decreases glucose levels in blood [37]. In the current study, despite similar THI levels (75–76) at both time points, cows produced higher MY (43 kg/d) at week 4 with lower glucose concentrations, while at week 11, MY decreased to 35 kg/d with higher glucose levels. This implies that the combination of heat stress and high milk production demand created a glucose deficit at week 4, while the natural decline in MY at week 11 allowed glucose recovery despite continued heat stress.

### Digestibility and nitrogen metabolism

There were no differences in DM, CP, and NDF digestibility between treatments. This agrees with Bahrami-yekdangi et al [6], who reported no changes in DM, CP, or digestibility when the dietary CP level was decreased from 16.4% to 14.8%. This implies that the reduction in dietary CP and RDP while increasing RUP did not negatively affect digestibility. Several studies have reported that lower dietary CP leads to less N excretion [31,38]. However, in the current study, there were no differences in total N excretion through feces and urine between treatments. We observed an improved NUE trend in HFSBM compared to SBM (25.05% vs. 22.79%; p = 0.08). Similarly, increased N efficiency has been reported when dietary protein is reduced [1,5]. This may be attributable to decreased CP and RDP and increased RUP through HFSBM supplementation. There were decreases in milk N, urine N, and total N excretion from week 3 to 11. The decrease in milk N is understandable as milk protein content naturally declined over the experimental period due to the lactation curve. For both urine N and total N excretion, although the reduction in feed intake was not statistically significant, likely due to limitations in indirect measurement, the numerical decrease in feed consumption during heat stress likely led to lower N intake, which in turn reduced N excretion. This implies that heat stress-induced reductions in nutrient intake influenced overall nitrogen metabolism, despite the lack of statistical significance in DMI measurements.

## CONCLUSION

The dietary SBM for dairy cows was replaced with HFSBM to reduce CP and RDP while increasing RUP. Although there were no differences on milk production, N composition, NUE (milk N/N intake) tended to be higher in the HFSBM group than in the SBM group. Overall, reducing CP and RDP tended to improve NUE without negatively affecting milk production or ruminal and blood parameters.

## Figures and Tables

**Table 1:** Chemical composition of experimental substrate feed (% dry matter [DM] basis)

	TMR^1)^	Concentrate I^2)^	Concentrate II^3)^	TMR-mix^4)^	Soybean meal	Heat-treated fermented soybean meal	Corn flake
Chemical composition (% DM)							
DM	64.92	88.79	88.61	88.76	88.84	91.07	85.91
Crude protein	14.40	23.13	23.70	18.0	52.60	56.62	9.50
Ether extract	4.48	3.68	5.35	3.70	1.09	1.64	4.10
Crude fiber	17.68	7.19	4.31	9.18	10.17	4.47	2.86
Ash	6.99	8.47	7.80	6.88	6.53	7.48	2.28
Acid detergent fiber	22.33	11.98	7.14	11.34	11.66	4.78	4.53
Neutral detergent fiber	40.79	27.91	17.85	30.21	20.14	35.82	15.55
Ca	0.91	1.30	1.15	0.80	0.39	0.39	0.47
P	0.40	0.75	0.60	0.59	0.59	0.77	0.26
Mg	0.28	0.29	0.26	0.17	0.28	0.34	0.11

1) Ingredients of TMR are listed in Table 2.

2) Ingredients: 18% of corn, 17% of corn gluten feed, 15% of palm kernel meal, 15.19% of soybean meal, 7% dried distiller’s grains with solubles, 5.76% of wheat bran, 5% of rapeseed meal, 4.37% of substrated wheat flour, 1.5% of soybean hulls, 2% of coconut meal, 2% of wheat grain, 2% of powdered fat (Calcium chloride) 2% of molasses, 1.2% of limestone, 0.43% of tricalcium phosphate, 0.1% of calcium sulfate, 0.3% sodium bicarbonate, 0.5% of biofeed, 0.4% processed salt, 0.1% vitamin-premix, 0.1% mineral-premix, 0.05% feed binder.

3) Ingredients: 17.72% of soybean meal, 16.4% of corn, 16.01% of corn gluten feed, 13% of substrated wheat flour, 12% of corn bran, 8% of whole soybean, 4% of wheat bran 2% of rice bran, 2% of corn gluten meal (60%), 1% of cocoa power, 1.23% of fat, 2.79 of powdered fat (calcium chloride), 1% of molasses, 1.25% of limestone, 0.4% sodium bicarbonate, 0.4% of processed salt, 0.1% of vitamin-premix, 0.1% of mineral-premix, 0.05% of sweeten, 0.15% of feed binder.

4) Ingredients: 23.88% of corn, 21% of corn gluten feed, 18% if palm kernel meal, 9% of soybean meal, 5.91% of dried distiller’s grain with solubules, 5.88% of soybean hulls, 4% of wheat bran, 2% of rapeseed meal 2% of corn germ meal, 1% of wheat grain, 0.3% of powdered fat (calcium chloride), 2% of molasses, 1.1% of limestone, 0.43% of tricalcium phosphate, 0.2% of calcium sulfate, 0.3% of sodium bicarbonate, 0.5% of biofeed, 0.5% of processed salt.

TMR, total mixed ration.

**Table 2:** Ingredients and chemical composition of the experimental diets

Ingredients	Diet	

	SBM	HFSBM
TMR ingredients (%DM)		
TMR-mix^1)^	29.65	29.96
Oat hay	12.29	12.42
Alfalfa hay	1.66	1.68
Corn flake	10.56	10.67
Timothy	5.30	5.36
Tall fescue	8.66	8.75
Cottonseed	0.65	0.66
Flaxseed	3.47	3.51
Protected fat	9.12	9.21
Sodium bicarbonate	0.63	0.63
Limestone	0.31	0.32
Vitamin premix^2)^	0.08	0.08
Concentrate I (%DM)^3)^	8.91	8.57
Concentrate II (%DM)^4)^	5.66	5.49
Soybean meal (%DM)	3.05	-
Heat-treated soybean meal (%DM)	-	1.53
Corn flake (%DM)	-	1.16
Total (%DM)	100	100
Chemical composition (%DM)		
DM (%)	71.74	71.55
NE_L_ (Mcal/kg DM)	1.69	1.69
CP (%DM)	16.87	16.24
RUP (%CP)	35.04	37.81
RDP (%CP)	64.96	62.19
RUP (%DM)	5.91	6.14
RDP (%DM)	10.96	10.1
EE (%DM)	4.45	4.51
Ash (%DM)	15.7	15.61
CF (%DM)	7.15	7.11
NDF (%DM)	37.86	38.2
ADF (%DM)	20.24	20.15
NFE (%DM)	55.96	56.65
NFC (%DM)	25.26	25.57
iNDF (%DM)	28.82	29.02
Ca (%DM)	0.96	0.96
P (%DM)	0.45	0.44
Mg (%DM)	0.28	0.28

1) Ingredients are listed in Table 2.

2) Composition: 3,500,000 IU/kg of Vitamin A,700,000 IU/kg of Vitamin D_3_, 2 g/kg of Vitamin E, 2 g/kg of Fe, 16 g/kg of Zn, 20 g/kg of Mg.

3),4) Ingredients are listed in Table 2.

SBM, soybean meal; HFSBM, heat-treated fermented soybean meal; TMR, total mixed ration; DM, dry matter; NE_L_, net energy of lactation; CP, crude protein; RUP, rumen undegradable protein; RDP, rumen degradable protein; EE, ether extract; CF, crude fiber; NDF, neutral detergent fiber; ADF, acid detergent fiber; NFE, nitrogen free extract; NFC, non-fiber carbohydrates; iNDF, indigestible NDF; Ca, calcium; P, phosphorus; Mg, magnesium.

**Table 3:** Effects of dietary replacement of soybean meal with heat-treated soybean meal on milk yield and milk compositions in Holstein lactating cows

Item	Treatment		SEM	p-value		
	
	SBM	HFSBM		Treatment	Period	Interaction
DMI (kg/d)	30.25	30.34	0.80	0.96	0.17	0.92
Milk yield (kg/d)	38.04	38.02	1.01	1.00	<0.01	0.98
FCM (kg/d)^1)^	36.84	35.49	1.01	0.65	<0.01	0.01
ECM (kg/d)^2)^	38.50	38.02	1.01	0.88	<0.01	0.37
FPCM (kg/d)^3)^	36.05	35.57	0.94	0.87	<0.01	0.38
Milk yield/DMI (kg/kg)	1.25	1.26	0.03	0.97	0.01	0.99
FCM/DMI (kg/kg)	1.21	1.18	0.03	0.71	0.05	0.05
ECM/DMI (kg/kg)	1.27	1.27	0.03	0.99	<0.01	0.61
FPCM/DMI (kg/kg)	1.19	1.18	0.03	0.97	<0.01	0.38
Fat (%)	3.69	3.64	0.09	0.90	0.01	0.16
Fat (kg/d)	1.37	1.41	0.05	0.83	<0.01	0.48
Protein (%)	2.88	2.85	0.04	0.78	<0.01	0.98
Protein (kg/d)	1.08	1.07	0.03	0.81	<0.01	0.01
Lactose (%)	4.82	4.81	0.02	0.92	0.07	0.70
Lactose (kg/d)	1.83	1.83	0.05	0.98	<0.01	0.97
Cells (×10^3^/mL)	52.58	59.19	3.85	0.67	0.03	0.05
BHBm (mmol/L)	0.08	0.03	0.01	0.14	0.17	0.33
SNF (%)	8.37	8.33	0.04	0.82	<0.01	0.63
MUN (mg/dL)	14.99	14.34	0.35	0.32	<0.01	0.88

1) FCM = 0.4×Milk yield+16.23×Fat yield (kg/d).

2) ECM (kg/d) = 0.323×Milk yield (kg/d)+12.82×Milk fat yield (kg/d)+7.13×Milk protein yield (kg/d).

3) FPCM = (0.377 + 0.116×milk fat [%]+0.06×milk protein [%])×Milk yield ([kg/d]).

SBM, soybean meal; HFSBM, heat-treated fermented soybean meal; SEM, standard error of the mean; DMI, dry matter intake; FCM, fat-corrected milk; ECM, energy-corrected milk; FPCM, fat- and protein-corrected milk; Cells, somatic cell; BHBm, milk beta-hydroxybutyrate; SNF, solids-non-fat; MUN, milk urea nitrogen.

**Table 4:** Effects of dietary replacement of soybean meal with heat-treated soybean meal on ruminal characteristics at 3 h after feeding in Holstein lactating cows

Item	Treatment		SEM	p-value		
	
	SBM	HFSBM		Treatment	Period	Interaction
pH				0.83	0.96	0.90
Week 4	6.91	6.98	0.07			
Week 11	6.95	6.93	0.09			
Ammonia (mg/dL)				0.42	0.63	0.18
Week 4	10.71	7.19	0.89			
Week 11	7.34	9.41	1.01			
Total VFA (mM)				0.11	0.16	0.16
Week 4	73.69	72.65	3.07			
Week 11	55.82	78.74	3.95			
Acetate (%)				0.23	0.13	0.12
Week 4	50.39	49.21	0.65			
Week 11	45.76	49.96	1.11			
Propionate (%)				0.30	0.96	0.44
Week 4	15.97	17.24	0.53			
Week 11	16.43	16.74	0.42			
Iso-butyrate (%)				0.17	0.13	0.27
Week 4	6.03	5.91	0.23			
Week 11	7.38	5.77	0.34			
Butyrate (%)				0.30	0.95	0.98
Week 4	12.92	12.73	0.12			
Week 11	12.94	12.73	0.15			
Iso-valerate (%)				0.14	0.13	0.28
Week 4	7.50	7.12	0.26			
Week 11	8.99	7.03	0.39			
Valerate (%)				0.14	0.14	0.14
Week 4	7.42	7.57	0.26			
Week 11	9.19	7.09	0.40			
Acetate: propionate				0.64	0.02	<0.01
Week 4	3.14	2.90	0.09			
Week 11	2.85	2.95	0.09			

SBM, soybean meal; HFSBM, heat-treated fermented soybean meal; SEM, standard error of the mean; VFA, volatile fatty acid.

**Table 5:** Effects of dietary replacement of soybean meal with heat-treated soybean meal on blood metabolites in Holstein lactating cows

Item	Treatment		SEM	p-value	
	
	SBM	HFSBM		Treatment	Period
Albumin (g/dL)					
0 h	3.72	3.71	0.04	0.94	0.25
3 h	3.81	3.82	0.04	0.89	0.43
ALT (U/L)					
0 h	29.77	26.60	0.97	0.20	0.08
3 h	29.00	26.86	1.07	0.47	0.09
AST (U/L)					
0 h	101.66	102.48	5.28	0.95	0.24
3 h	100.00	101.62	5.17	0.90	0.06
Glucose (mg/dL)					
0 h	40.31	41.50	2.77	0.67	<0.01
3 h	36.50	35.17	3.04	0.92	0.38
BUN (mg/dL)					
0 h	17.68	17.63	0.60	0.97	<0.01
3 h	19.92	18.77	0.53	0.19	<0.01
Total protein (g/dL)					
0 h	7.24	7.30	0.06	0.61	0.96
3 h	7.32	7.56	0.05	0.03	0.76
Calcium (mg/dL)					
0 h	9.38	9.81	0.11	0.02	0.33
3 h	9.06	9.58	0.11	0.03	0.09
Inorganic phosphate (mg/dL)					
0 h	6.11	5.91	0.19	0.68	0.91
3 h	5.52	5.62	0.17	0.78	0.37
Magnesium (mg/dL)					
0 h	2.22	2.24	0.03	0.74	0.25
3 h	2.32	2.29	0.04	0.76	0.22

Treatment and period interactions were not significant (p>0.05) and were removed from the model.

SBM, soybean meal; HFSBM, heat-treated fermented soybean meal; SEM, standard error of the mean; ALT, alanine aminotransferase; AST, aspartate aminotransferase; BUN, blood urea nitrogen.

**Table 6:** Effects of dietary replacement of soybean meal with heat-treated soybean meal on N intake and excretion in Holstein lactating cows

	Treatment		p-value			

	SBM	HFSBM	SEM	Treatment	Period	Interaction
Digestibility (%)						
DM	56.46	57.48	0.77	0.53	0.89	0.19
CP	58.51	57.75	0.92	0.70	0.67	0.54
NDF	47.94	50.64	1.04	0.20	0.31	0.07
N intake (g/day)	815.87	787.27	18.96	0.48	0.18	0.92
Excretion						
Fecal output (kg/day)	13.16	12.94	0.44	0.82	0.26	0.47
Urine output (L/day)^1)^	44.32	46.63	1.78	0.57	0.97	0.76
Urine creatinine (mg/dL)	43.08	42.36	2.01	0.87	0.76	0.31
Urine nitrogen (%)	0.78	0.75	0.04	0.64	0.10	0.82
Milk N (g/day)	185.91	196.74	6.26	0.45	<0.01	0.91
Urine N (g/day)	360.45	336.59	10.36	0.29	<0.01	0.04
Fecal N (g/day)	337.55	334.38	11.63	0.90	0.52	0.68
N excretion (g/day)^2)^	698.00	670.96	17.14	0.53	0.01	0.46
Nitrogen use efficiency (%)^3)^	22.79	25.05	0.64	0.08	0.06	0.89

Fecal DM (kg) = DM intake×(1–DM digestibility).

Indigestible neutral detergent fiber (iNDF) contents in feeds, refusals, and feces were used to estimate fecal excretion of DM.

1) Urine output was estimated at 29 of the coefficients (urinary creatinine excretion, mg/d per kg of BW).

2) N excretion = Urine N+Fecal N.

3) N-use efficiency = 100×milk N/intake N.

N, nitrogen; SBM, soybean meal; HFSBM, heat-treated fermented soybean meal; SEM, standard error of the mean; DM, dry matter; CP, crude protein; NDF, neutral detergent fiber.

## Data Availability

Upon reasonable request, the datasets of this study can be available from the corresponding author.
